# 3D Localization of Hydrating Sources in Concrete Based on AE and Tomography

**DOI:** 10.3390/s26041345

**Published:** 2026-02-20

**Authors:** Eleni Korda, Fuzhen Chen, Hwa Kian Chai, Geert De Schutter, Dimitrios G. Aggelis

**Affiliations:** 1 Department of Mechanics of Materials and Constructions, Vrije Universiteit Brussel, 1050 Brussels, Belgium; dimitrios.aggelis@vub.be; 2 Department of Structural Engineering and Building Materials, Ghent University, 9052 Gent, Belgium; geert.deschutter@ugent.be; 3 Institute for Infrastructure and Environment, School of Engineering, University of Edinburgh, Edinburgh EH9 3FG, UK; f.chen-27@sms.ed.ac.uk (F.C.); hwakian.chai@ed.ac.uk (H.K.C.)

**Keywords:** acoustic emission, 3D localization, travel-time tomography, superabsorbent polymers, hydration

## Abstract

Plastic shrinkage and self-desiccation, along with the associated early-age cracking, are still among the most important factors that influence long-term performance of concrete structures, including durability. Superabsorbent polymers (SAPs) have been widely researched for application in concrete to mitigate shrinkage through facilitating effective internal curing by releasing water into the mixture to promote continuous hydration of cement. The acoustic emission (AE) monitoring technique, due to its high sensitivity, has proven very effective in tracking the process of water release by SAPs in concrete during early-stage curing. Typically, AE parameters such as cumulative activity, amplitude and energy are utilized to characterize the kinetics of curing processes. While these parameters indicate well the internal activity of SAPs in time, they do not offer information on the precise location of the active sources within the material’s volume, leaving a crucial gap in the understanding of the ongoing microstructural changes caused by internal water distribution and cement hydration. In this sense, AE event source localization can offer information about the active zones of water hydration activity in the material 3D domain, allowing detection of their evolution during concrete curing. Meanwhile, Acoustic Emission Tomography (AET) computes ultrasonic velocity distributions in different periods of monitoring, which are governed by acoustic characteristics of the concrete mixtures, to visualize material stiffness development spatially and temporally. This level of insight is particularly important for SAP concrete, where uniformity of internal water curing is essential for ensuring long-term durability and material soundness. By visualizing how the hydration sources evolve in real time, these methods offer an effective, non-destructive, and cost-effective solution for early-age concrete quality control, which would be challenging to achieve through other techniques.

## 1. Introduction

Acoustic emission (AE) is a robust non-destructive technique that detects internal material processes by recording the release of elastic waves. Due to its high sensitivity, AE has been increasingly utilized over the past decade to monitor fresh cementitious materials, where complex physico-chemical processes occur simultaneously during the hydration reaction [1,2,3].

Source localization is a key feature of AE, where the coordinates of a source (AE “event”) are determined from differences in elastic wave arrival times at multiple sensors [1]. Depending on dimensionality (1D, 2D, or 3D), a minimum of two, three, or four sensors is required. Once the wave velocity in the material is known, the source location can be estimated using these time differences and the known sensor positions. The arrival time corresponds to the onset of the P-wave, which is the first detectable disturbance, making onset determination crucial for localization accuracy. Arrival time can be obtained manually or automatically through a picking algorithm [4]. Localization accuracy is further influenced by velocity variations within the travel path, scattering induced by material heterogeneity, and a low signal-to-noise ratio [4].

Accurate localization allows for a quantitative AE analysis of a process/damage evolution. Several localization techniques exist, such as modal acoustic emission, neural networks [5,6,7], beamforming [8,9], and triangulation [1]. Conventional AE analysis assumes a homogeneous, isotropic medium, but in heterogeneous or anisotropic materials, or where boundary conditions alter the wave propagation, material properties and complex ray paths must be considered more carefully. Coupling AE localization with AE tomography (AET) [10] addresses these limitations by accounting for velocity variations and nonlinear wave paths, leading to significantly more accurate event coordinates.

In fresh and early-age concrete, 3D localization characterizes not only damage but also the mechanisms of curing and cracking. While these processes are effectively captured as temporal trends in the cumulative AE activity, their spatial distribution is not straight-forward. Source localization clarifies this spatial intensity, which is particularly critical when evaluating novel concrete systems like superabsorbent polymer (SAP) concrete [11,12]. Such admixtures have recently been used to mitigate early-age shrinkage caused by low water-to-cement (w/c) ratios. SAPs absorb water during mixing and release it during self-desiccation, providing internal curing [13], counteracting rapid water consumption and, hence, shrinkage. However, their swollen state in the freshly mixed material results in macropores in the hardened cementitious matrix, which complicates wave propagation [14]. Localizing AE sources linked to SAP activity helps track internal curing evolution and homogeneity, particularly under drying conditions where SAP behavior differs from sealed systems, and aids in interpreting hydration microstructurally.

Despite its importance, source localization in fresh concrete has been scarcely addressed due to evolving wave speed and strong attenuation that limits source detection by multiple sensors. Most 3D localization and tomography studies focus on hardened concrete, investigating cracking [15], corrosion [16,17], self-healing [18], spalling [19], and damage under loading [20,21,22,23]. Early-age studies have mainly targeted cracks from thermal or shrinkage stresses [2,24], typically using 1D localization. Some researchers examined curing and velocity evolution through Pencil Lead Break (PLB)-based localization [3], or 3D AE localization during early drying shrinkage from 24 h of curing onwards [25].

This study advances the state of the art through three primary contributions: (i) the implementation of 3D AE localization during the first minutes of hydration, providing a first-time view of the early-stage activity; (ii) the development of a nonlinear travel-time tomography model that accounts for spatial and temporal velocity variations between the inhomogeneous fresh concrete and the metal formwork; and (iii) the characterization of the spatial evolution of internal curing, mapping the evolution of SAP activity into deeper zones. By integrating iterative source localization with tomographic inversion, this work successfully localizes the internal hydrating sources, while excluding signals arising from background noise and geometric artifacts.

## 2. Materials and Methods

### 2.1. Compositions

The mixture investigated contained Portland-type cement CEM I 52.5 N Strong (Holcim, Belgium), and the composition was made with a low effective w/c ratio, equal to 0.35, making it prone to autogenous shrinkage and suitable for SAP addition. Tap water was utilized for mixing. For aggregates, coarse gravel stones, fine gravel stones and river sand were added in proportions of 2.36:1.27:1.27, respectively, with respect to cement mass. To ensure sufficient workability, superplasticizer (MasterGlenium 51, BASF, Germany) was added at a percentage of 0.6 m% by cement mass, denoted as SP. The mixture contained SAPs in a content of 0.2 m% (per mass of cement). Since SAPs absorb part of the available water during mixing by swelling, additional water was used to compensate for the loss in workability, equal to 20 g/g SAP [14]. The extra amount of water is theoretically entrained in the SAPs and is gradually released during the hydration reaction, resulting in a mixture with an effective w/c ratio of 0.35. The sand/aggregate ratio was also 0.35. The slump value was equal to 3 cm, assessed according to EN12350-2: Testing fresh concrete - Part 2: Slump test. Brussels: European Committee for Standardisation, Brussels, 2019. The proportioning of the composition is shown in Table 1.

### 2.2. Specimens

**Figure 1:**
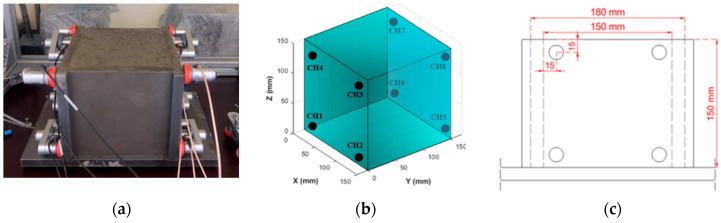
(**a**) AE monitoring setup of concrete cube, (**b**) AE sensor localization layout (homogeneous model) and (**c**) AE sensor positioning.

This study mainly focuses on investigating the active curing zones of SAPs. The two specimens investigated, denoted as SAP1 and SAP2, were in the form of 150 mm concrete cubes prepared using a laboratory concrete mixer of a 20 L capacity, where the material was mixed at a speed of 107 rpm. The total mixing time was four minutes: one minute of mixing the dry components, including SAPs, followed by three minutes of mixing with water. The material was then poured into the steel formwork in two layers and vibrated for 30 s after each layer was poured. The metallic nature of the formwork allowed for sensitive AE signal acquisition, along with secure attachment of the AE sensors on its surfaces through magnetic clamps, as shown in Figure 1a. The formwork has internal dimensions of 150 mm and a thickness of 15 mm, as shown in Figure 1c. Due to the lower wave attenuation by steel compared to the fresh and hardening concrete mixture, the formwork acts as a waveguide, thus facilitating good sensitivity of AE signal detection in the highly viscous fresh state of concrete, and potentially mitigating loss of any meaningful AE events by attenuation. The selected SAP type yielded a high number of AE hits and events, due to its large size and subsequent detachment from the cementitious matrix, as will be explained later, which is crucial for AE source localization and tomographic reconstruction. The monitoring period lasted for approximately three days, and the specimen surface was unsealed and exposed to ambient conditions (T = 20 °C ± 2, RH = 55–60%). The typical 28-day compressive strength of the specimens was, on average, 67 MPa. A reference specimen (REF) with an identical base composition, but no SAPs or additional SAP water, was monitored via AE to compare the cumulative AE activity. Tomographic imaging was not performed on this specimen.

### 2.3. Acoustic Emission Monitoring and Source Localization

The cubes were monitored using eight AE sensors attached to the formwork at 15 mm from each corner of the formwork’s internal dimensions, as shown in Figure 1. The utilized sensors were R15-α, high-sensitivity, piezoelectric transducers with 150 kHz resonance frequency, while the Micro-II express (Physical Acoustic Corporation, NJ, USA) acquisition system was used for data acquisition. The physical and chemical processes taking place in the fresh/hardening material generate signals that propagate as elastic waves and are received by the sensors once they reach the material’s surface. The piezoelectric elements then transform the transient pressure changes into electric waveforms which are amplified by 40 dB, using 1220A preamplifiers, before being digitized in the acquisition board with a sampling rate of 1 Mega-Samples-Per-Second (MSPS). In the latter, the waveform parameters are analyzed, and the waveforms are displayed digitally. A low threshold of 35 dB was selected to filter out laboratory noises, while preserving the desired sensitivity.

Three-dimensional AE source localization was performed using the AEWin (Physical Acoustic Corporation, West Windsor Township, NJ, USA, https://www.physicalacoustics.com/byproduct, accessed on 4 January 2026) software [26], which captures, stores, and processes the recorded waveforms. Localization is based on a trilateration approach, which requires arrival time information from multiple sensors to estimate the source coordinates and arrival time [18,27]. While at least four sensors are theoretically sufficient for 3D localization, in this study, a minimum of six sensors was selected for an AE event to be formed, in order to improve the robustness of the results.

Hits detected independently on each sensor are grouped into a single AE event if they occur within a predefined time window (peak definition time, hit definition time, hit lockout time, overcall value) consistent with wave propagation across the specimen. Arrival time picking on each sensor is obtained automatically as the first threshold crossing of the recorded waveform. No preprocessing (e.g., filtering or smoothing) was applied to the waveforms. The time of the first recorded hit within an event is used as a reference, and the relative arrival time delays of the subsequent hits are computed accordingly.

Source localization is then formulated as an inverse time-of-arrival problem, in which the unknown parameters are the source spatial coordinates (x,y,z) and the event origin time t. Assuming that the P-wave arrival times have been correctly identified on a group of N sensors, the arrival time at the *i*th sensor can be expressed as a first-order approximation [10]:

(1) $${t_{i}}^{A}=\;\frac{\sqrt{{\left({x_{i}}^{S}-x\right)}^{2}+\;{\left({y_{i}}^{S}-y\right)}^{2}+\;{\left({z_{i}}^{S}-z\right)}^{2}}}{c_{i}}+t$$

where ${{(x}_{i}}^{S},\;{y_{i}}^{S},\;{z_{i}}^{S}$) is the known coordinates of the ith sensor, (x, y, z) is the unknown location of the AE source to be determined, $c_{i}$ is the effective wave velocity between the source and the ith sensor, and *t* is the unknown arrival time of the event defined relative to the first threshold crossing of the first hit in the event. In AEwin, the inverse problem is solved and refined by minimizing the residuals between the measured and calculated arrival times using a Simplex optimization fitting algorithm [28]. A single effective wave velocity is assigned to compute the events for each examined curing stage. Localization time-window parameters were selected based on prior calibration experiments and are consistent with values reported in [29,30]. The test parameters and sensor configuration are summarized in Table 2 and Table 3, respectively.

Equation (1) is based on a straight-ray approximation for wave propagation. In practical applications, and in the AE localization part of this study, a homogeneous wave velocity is assumed for all propagation paths. The sensor positions were assumed to be directly on the concrete specimen, while the presence of the formwork was neglected.

Considering that concrete undergoes hydration and stiffness increases, the elastic wave velocity changes as well. This makes the localization complicated. To overcome this issue, representative velocities were used to account for each curing stage and changing stiffness. The utilized P-wave velocity is obtained by UPV measurements obtained by the commercially available Freshcon device [31], and the experimental setup is described in previous research [14]. UPV results are shown in Section 4.1. The three-day monitoring period was divided into three phases according to the curing stages of interest, namely early hydration, internal curing and hardening. The velocity is calculated by averaging the UPV measurements over the relevant period and weighing them based on the areas of the steel formwork and concrete within the cross-section. After the 3-day acquisition was completed, the AE data was analyzed in the AEWin software by considering the respective velocities per curing stage. The analysis was followed by filtering out the resulting events that did not correspond to the identified curing timeframe. The internal curing period was selected as the timeframe where SAPs were the most active (12–20 h), as demonstrated by the AE activity. The curing stages and corresponding velocities can be found in Table 4.

### 2.4. Acoustic Emission Tomography

#### 2.4.1. Principles of AET and Integration with Velocity Distribution Modeling

In acoustic tomography, an image of variations in a physical property within an object is generated by analyzing data from acoustic waves transmitted through the object at multiple angles. These variations may include attenuation or wave velocity in transmission tomography or acoustic impedance mismatches in reflection tomography [10]. In conventional acoustic tomography, elastic waves are generated by multiple external sources and recorded by receivers positioned at different locations around the specimen. The principle of AE tomography is that the velocity variations inside the material are obtained using unknown random locations obtained by localized sources within the material [10]. These internal sources act as the trigger points emitting the waveforms that are subsequently recorded by the sensors.

The procedure is divided into three main computational steps: (1) initial source localization, in which tentative AE source positions are estimated from the recorded arrival times using an initial velocity model; (2) forward-problem solving, which computes the elastic wave arrival times at predesignated nodes (here, the position of the AE sensors); and (3) inverse-problem solving, in which elastic wave velocities are updated (under the first-arrival travel-time formulation) to minimize the misfit between the observed and predicted arrival times. The arrival time computation is directly affected by the acoustic properties of the material, which in turn affects the computation of the elastic wave velocity. The last two parts are alternated iteratively to obtain the velocity distribution in the examined structure: after each inversion update of the velocity field, the AE sources are re-localized, and the forward travel times are recomputed. The model parameters are the material velocity field and the source locations.

To assess the influence of the steel formwork on 3D localization and the impact of SAPs on wave-velocity development, an enhanced AEΤ methodology is employed. While traditional AE(T) localization, described previously, assumes isotropic properties and linear paths, the acoustic impedance mismatch between fresh concrete and a stiff steel formwork results in significant nonlinearities. To mitigate these inaccuracies, this part of the study utilizes the AET method proposed by Chen et al. [32]. Specifically, the method employs velocity distribution modeling (VDM) to account for the changes in elastic wave propagation induced by the multi-material configuration (fresh concrete and stiff steel formwork). This adoption improves source localization in complex multi-material configurations and yields AET results that are more physically consistent. Elastic wave source localization is integrated with travel-time tomographic reconstruction to capture the velocity distribution variations. Using a geometry and sensor configuration identical to the setup in Section 2.3, AET simultaneously computes source locations while refining the 3D velocity field. Unlike conventional methods that assume homogeneity and linear travel paths, this methodology employs an inhomogeneous model to account for the steel formwork and the resulting velocity mismatch between materials, more accurately simulating the 3D wave behavior.

#### 2.4.2. Computational Model and Random Event Acquisition (Initial Source Localization)

**Figure 2:**
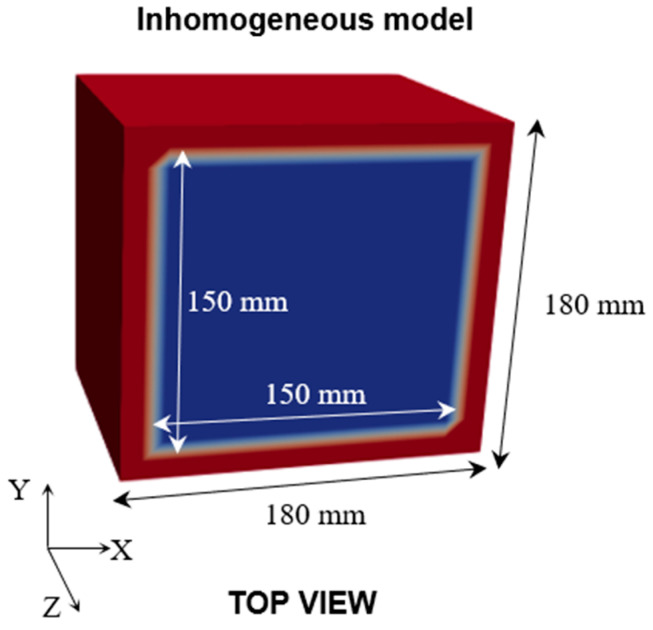
Finite-difference model (FDM) adopted for the AE source localization and tomographic reconstruction computations. The blue area represents concrete, while the red area represents the steel formwork of 15 mm thickness.

A 3D Finite Element Model (FEM) was initially developed to represent the specimen’s geometry and to define the concrete and steel formwork domains. A Cartesian grid with a spacing of 5 mm was then constructed for finite-difference computations, onto which the FEM-defined material domains were mapped. Subsequently, the VDM was assigned to the finite-difference grid (based on the FEM-defined material domains) to account for the heterogeneous complexity resulting from the steel formwork. This configuration, referred to as the finite-difference model (FDM) in Figure 2, is essential for simulating realistic elastic wave propagation; all forward travel-time calculations and the subsequent inversion were performed on the FDM. Unlike conventional forward modeling methods that assume material homogeneity, the VDM approach assigns different velocity characteristics to different regions of the FDM to represent the medium’s heterogeneity more accurately. The model was then used to perform 3D localization and velocity reconstruction through AET. For each curing stage listed in Table 4, the average P-wave velocity was used as the background velocity for the concrete region, while the steel formwork was assigned a higher velocity (5800 m/s) consistent with its elastic properties.

AE hits obtained during the acquisition, suitable for AE tomography analysis, were extracted using a Python Version 3.11script. The first threshold crossing was adopted to determine the arrival times. The selection of signals was based on the following criteria:

1. Each valid AE event was independently detected by eight different sensors, ensuring reliability in the data acquisition process.
2. A signal was considered valid only if all sensors recorded it within a very short time window Δt. Δt was set as a conservative upper bound derived from the maximum physically plausible source–sensor path and the stage-dependent minimum expected P-wave velocity, yielding Δt equal to approximately 100 µs, 74 µs, and 70 µs for the 0–12 h, 12–20 h, and 20–73 h intervals, respectively. A modest safety margin was included to account for picking uncertainty and residual heterogeneity. These time differences were estimated using a weighted average of the elastic wave propagation velocity.

3D source localization was performed using the time difference of arrival (TDOA) method [33]. To ensure data integrity, an explicit event association protocol was applied: only events triggering all eight channels within a stage-dependent coincidence window Δt were retained. This strict selection mitigates noise, spurious hits, and event misassociation. In total, 233, 2048, and 2196 events satisfied these criteria for the 0–12 h, 12–20 h, and 20–73 h intervals, respectively. These events provide the sufficiently dense coverage required for AET, with the 8-channel trigger requirement ensuring the reliability of the input arrival times.

#### 2.4.3. Forward and Inverse Problems

##### Forward Problem: Physical Formulation

The forward problem in AET requires simulation of wave propagation to obtain theoretical arrival times. Numerous numerical schemes exist for computing arrival times in complex media [34]. In civil engineering, propagation has often been simplified by assuming linear paths, though such straight-ray approximations do not represent strongly heterogeneous media like the concrete–steel system. To account for strong heterogeneity and nonlinearity, wave propagation is modeled using a full wavefront method. This approach has been widely used in geotechnical problems. Under the high-frequency (first-arrival) approximation, the travel-time field T(x) satisfies the eikonal equation [35], a nonlinear first-order PDE governing the first-arrival wavefront propagation, as follows:

(2) $$∥\nabla T(x)∥=\frac{1}{v(x)}$$

with the source condition $T\left(x_{s}\right)=0$ at the current event location $x_{s}$, where v(x) is the spatially varying P-wave velocity and travel times are evaluated at the sensor nodes. In a 3D Cartesian coordinate system, this relationship is expressed as

(3) $$\sqrt{{\left(\frac{\partial T}{\partial x}\right)}^{2}+{\left(\frac{\partial T}{\partial y}\right)}^{2}+{\left(\frac{\partial T}{\partial z}\right)}^{2}}=\frac{1}{v(x)}$$

where x, y, and z are the Cartesian coordinates.

##### Forward Problem: Numerical Solution

The forward problem is solved via full wavefront tracking using the Fast Marching Method (FMM), which explicitly rejects the straight-ray assumptions that are commonly adopted in traditional AE localization. Because the eikonal equation cannot be solved analytically in heterogeneous 3D domains, FMM is utilized to compute theoretical arrival times by simulating the wavefront propagation through the discretized FDM grid. This approach is very stable for the high-velocity mismatch of the concrete–steel interface [35,36]. By implementing an upwind finite-difference scheme, the model preserves causality and accurately captures the nonlinear propagation paths necessary for a reliable inverse reconstruction. As this is a travel-time formulation (not a time-domain elastodynamic simulation), no explicit time stepping is involved; the computational domain is defined by the 5 mm Cartesian grid covering the specimen and the steel formwork.

Here, heterogeneity is explicitly represented by VDM: the computational grid distinguishes the steel formwork and the concrete volume, using measured/assigned background velocities as initial values (5800 m/s for the steel and varying velocities for the curing concrete), which are subsequently updated during the inversion process.

##### The Inverse Problem

Tomographic inverse-problem solving typically relies on algebraic reconstruction methods such as SART [37] or SIRT [38]. Although these methods can solve linear systems, they cannot address the nonlinear eikonal formulation. Therefore, a quasi-Newton algorithm from the least-squares optimization family was employed here, as established by Brantut [37]. The quasi-Newton method is selected because it offers superior performance in 3D configurations [39,40,41]. Because the event origin time is unknown, the inversion is formulated using TDOA; i.e., arrival times are referenced to the first-arriving sensor for each event, and the misfit is evaluated between observed and predicted TDOA values. The model parameters are the cell-wise velocity (or slowness) field on the fixed 5 mm FDM grid and the AE source locations (updated through re-localization after each velocity update). Accordingly, the velocity field is updated by minimizing the misfit between observed and predicted arrival times (obtained from forward modeling) using the quasi-Newton iterative scheme as follows:

(4) $$m_{k+1}=m_{k}+\alpha_{k}\Delta m_{k}$$

where m contains the model parameters mentioned above, $\Delta m_{k}$ is the quasi-Newton search direction, and $\alpha_{k}$ is the step size. The objective function is the (optionally weighted) least-squares norm of the travel-time residuals. Unless stated otherwise, uniform weights are used because arrival times are picked through the first threshold crossing with comparable timing uncertainty across channels. Mild spatial smoothness damping is applied to suppress cell-to-cell oscillations in the updated velocity field. The step size α is selected to ensure a monotonic decrease in the residual. Iterations stop when the residual reduction between successive iterations becomes negligible (e.g., <1%) or when a maximum number of iterations is reached.

## 3. Results

### 3.1. Cumulative AE Activity

**Figure 3:**
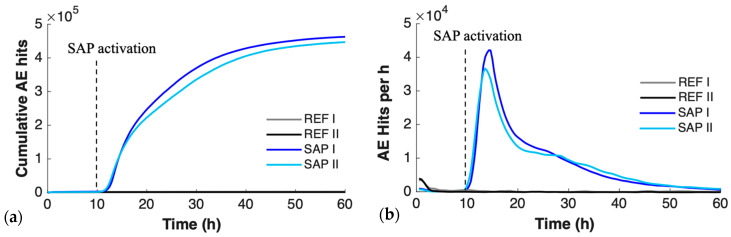
(**a**) Cumulative AE activity and (**b**) hit rate of REF vs SAP concrete. Numbers I and II represent two specimens.

The cumulative AE activity provides a first indication of the internal state of the material and aids in determining the AE signature of the different curing stages. When SAPs desorb their entrained water, they progressively shrink towards their original size and, therefore, detach from the internal surface of their cavity. This detachment, previously confirmed via scanning electron microscopy [42], is accompanied by a significant release of AE energy. This explains the pronounced AE response observed in the SAP concrete, a feature absent in the SAP-free (REF) concrete. Figure 3 shows that the REF curve almost coincides with the horizontal axis at this scale, highlighting the contrast. The onset of internal curing activity occurs at approximately 10 h, and it continues up to about 60 h of curing. Typical AE waveforms of the SAP activity can be found in the Discussion Section. AE can capture the internal curing with high precision and provide temporal insights into the curing state of the material in a non-destructive manner. At the same time, the increased AE activity of SAP concrete results in the formation of thousands of events, which is essential for localization and tomographic purposes.

### 3.2. AE Localization (Homogeneous Material Assumption)

**Figure 4:**
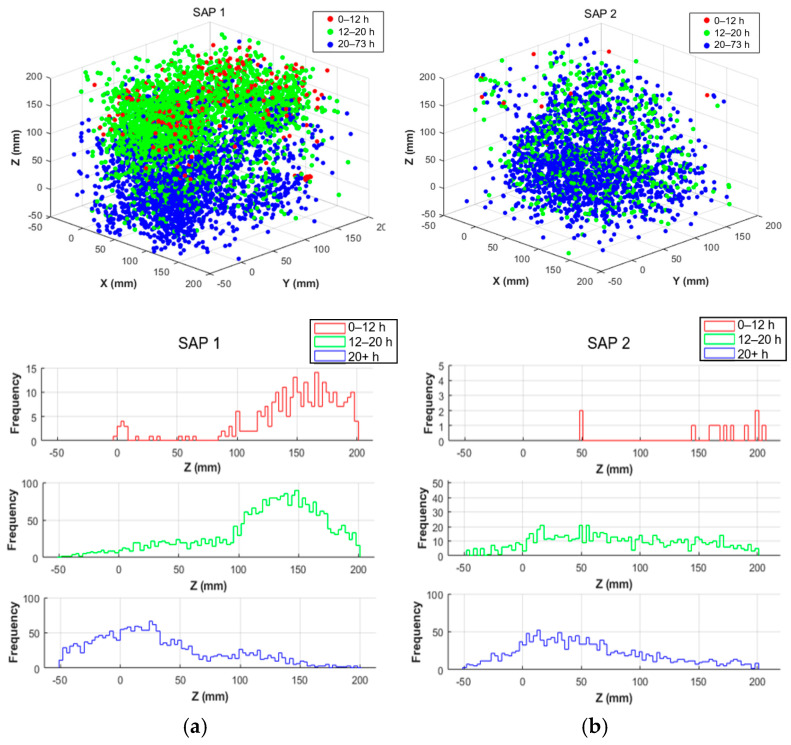
Temporal and spatial evolution of AE event localization in SAP concrete; 3D view and frequency distribution in Z axis for (**a**) SAP1 and (**b**) SAP2 specimens.

The detected events and their corresponding axial histograms are presented in Figure 4. The results reveal a very interesting and clear progression of AE sources from the surface towards the core of the specimen, demonstrating that SAP activation gradually advances into deeper zones as curing proceeds. During the initial curing stage (0–12 h), the AE sources are predominantly concentrated at the upper drying surface, peaking at approximately 150 mm, as seen by the red-line distribution plot for SAP1, while almost no activity is registered below the height of 100 mm in Figure 4a. At this stage, SAPs are not activated until after 10 h. Therefore, the majority of the sources arise from drying processes and initial SAP detachment, which starts at the surface. Bleed water, evaporation, and surface shrinkage can all be contributing factors, while SAPs close to the surface are reasonably activated first due to surface drying (towards the later 10–12 h timeframe). As curing proceeds, the AE sources are localized in the upper half of the formwork (12–20 h, green dots). At this stage, the main activity of SAPs takes place through release of most of their entrained water, while drying progresses due to the ongoing evaporation. SAPs start detaching from their pore walls (starting at the surface where drying is more pronounced), but also in the core of the specimen, where the progression of self-desiccation (also known as internal drying) accelerates the internal moisture consumption. Finally, beyond 20 h (blue dots), the AE sources are primarily localized in the lower half of the specimen, showing that SAPs at the bottom will activate last due to the higher available internal relative humidity (RH) levels of this protected (by the formwork) region. This is also shown in the bottom distribution plot, where the peak of the activity is found at 30 mm, with much lower activity concentrated above 50 mm.

For SAP2, shown in Figure 4b, a min/max of six sensors was used for source localization, to examine the possibility of capturing a similar trend using a lower minimum sensor number, and to compare the trends to SAP1, where a minimum of eight sensors was selected. The results show a similar trend to SAP1, with events progressively moving towards the bottom of the formwork, but fewer events are localized. This is a significant finding, not just for non-invasive monitoring of the SAP activity, but also for showing, for the first time, the transient development of the actual active zones of the SAPs in the concrete volume.

An important note here is that drying induces a gradient in internal curing homogeneity and acts on top of the self-desiccation mechanism, with the latter being the driving force for internal curing in sealed systems during the hydration reaction. In open systems, such as the one studied here, both mechanisms take place simultaneously, making the internal curing interpretation more complicated. Curing, drying and self-desiccation influence the SAP behavior differently. Self-desiccation is an internal global phenomenon across the volume, taking place as the hydration reaction “consumes” the available water to form the hydration products and the solid skeleton and therefore reduces the internal RH. In contrast, drying, which is external and surface-driven, results in more abrupt moisture loss and subsequent SAP detachment. From the progressive trend observed in the results, one can deduct that, in open systems, drying has a stronger impact on the volumetric and temporal activation of SAPs, while self-desiccation acts as a secondary but simultaneous process. More details on the hydration processes and SAP behavior are provided in previous research [43].

### 3.3. AE Tomography Localization (Inhomogeneous Material Assumption)

**Figure 5:**
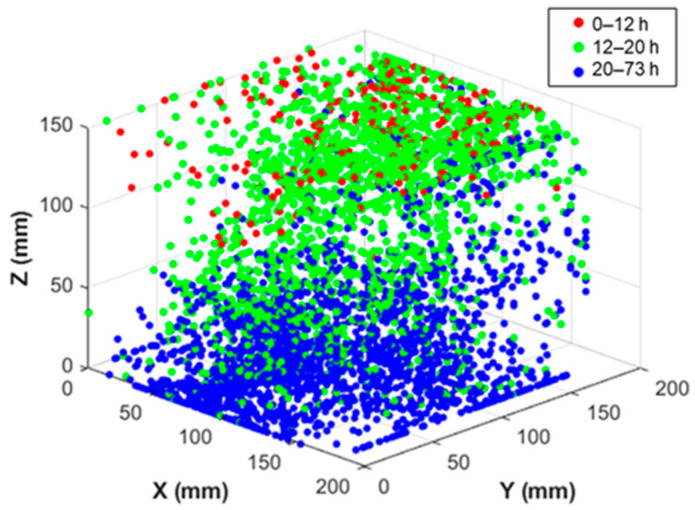
3D view of the temporal and spatial evolution of AE event localization in SAP concrete as obtained from AE tomography.

**Figure 6:**
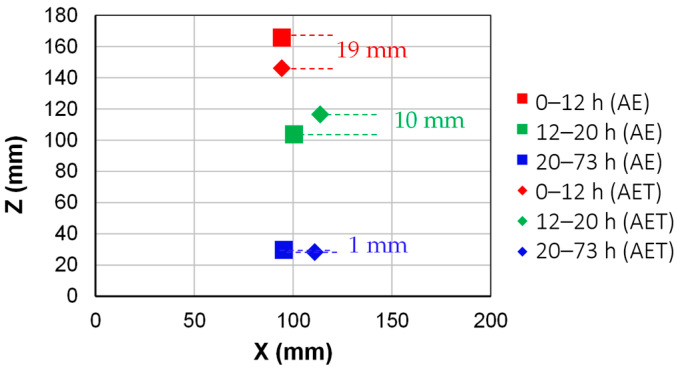
Comparison of XZ mean location coordinates per localization method: conventional AE localization and AE tomography localization. The results of AE localization have been derived using events localized by a min/max of 8 sensors to match the AET localization conditions.

The 3D localization results obtained through AET are discussed herein. By plotting events from all three curing stages in Figure 5, their evolution is shown to be similar to the one obtained by linear AE localization, which further supports the observation of the gradual progression of internal curing sources from the surface towards the bottom of the formwork. The number of localized sources is sufficient with AE tomography, which strengthens the localization reliability. Figure 6 shows the mean values of the coordinates obtained by the two different localization methods. SAP1 was used for the comparison, in which eight sensors were selected as a minimum for localization, to match AET. The comparison between the two approaches (AE-AET) shows slight discrepancies in the 2D and 3D mean coordinate values. In the 3D space, the average coordinate values differ by approximately 1 to 20 mm. From both approaches, one commonality can be derived: there is a significant shift in AE activity from the top to the center/bottom of the specimen. The significant findings show that the adopted methodologies allow non-invasive and simple monitoring of the kinetics of the desorption process that would otherwise be difficult to characterize and depend on several material and environmental parameters. 

**Figure 7:**
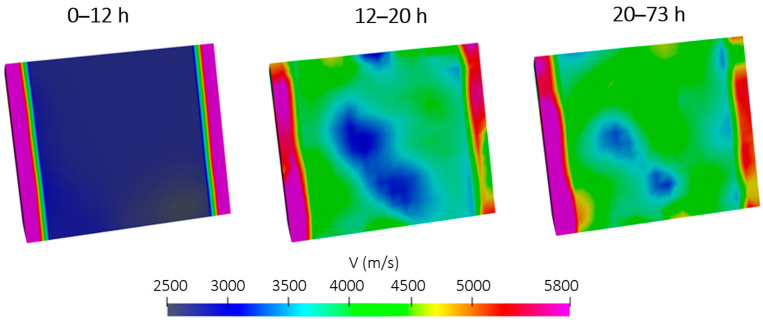
Velocity distribution tomograms in SAP concrete as obtained from AE tomography assuming an inhomogeneous steel formwork–concrete model.

The 3D velocity evolution is shown in Figure 7. During the initial stage (0–12 h), the interior of the specimen is dominated by low-velocity regions, indicating that the concrete matrix is still soft and only limited stiffness development has occurred. In the 12–20 h interval, the tomograms reveal a clear increase in velocity, with high-velocity zones progressively forming from the outer regions and extending towards the central and lower parts of the specimen. Low-velocity areas become confined mainly to the core and the bottom region, suggesting that these zones are still at an earlier stage of hydration or retain higher internal relative humidity. This pattern matches the AE-based observation that the most intense internal curing activity and water release occur during this period, with sources gradually migrating away from the surface towards deeper zones. Beyond 20 h, the velocity field became predominantly high throughout the volume, and only small pockets of relatively low velocity remained. This indicates that the concrete had largely hardened and that the stiffness distribution became more uniform, in agreement with the reduced AE activity associated with SAP desorption and detachment. The overall trend from a mostly low-velocity interior to an almost entirely high-velocity volume is strongly supported by the interpretation of a curing front progressing from the exposed surface towards the protected bottom, as inferred from the 3D AE localization results. These velocity reconstructions demonstrate that the AET results are physically consistent with the independent AE observations and with the expected evolution of stiffness in internally cured concrete.

## 4. Discussion

### 4.1. Measured Hydration-Related Processes

**Figure 8:**
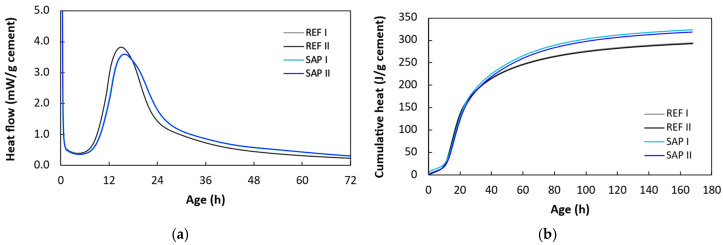
(**a**) Heat flow and (**b**) cumulative heat calculated from isothermal calorimetry measurements of cement pastes. The values are calculated with respect to the mass of the binder, here being the cement. Measurements were conducted at 20 °C. Numbers I and II represent two different specimens.

The hydration reaction and its evolution control the rate at which SAPs release their stored internal curing water, as this release is governed by the evolving water demand of the cement matrix. Figure 8 illustrates the heat flow and cumulative heat release during hydration obtained from isothermal calorimetry measurements (closed system) on cement pastes of the REF and SAP compositions. Owing to the higher total water content in the SAP composition, the initial hydration rate is lower compared to the REF mixture. However, after approximately 7 days, the SAP mixtures exhibit a higher cumulative heat release, eventually leading to a higher degree of hydration. The behavior reflects the beneficial effect of internal curing, which promotes continued hydration at later ages. From the calorimetry curves, one can also distinguish the hydration stages selected in this paper. The period 0–12 h corresponds to the early hydration stage, 12–20 h mainly to the acceleration period, where the reaction rate is highest, and 20+ h to the later deceleration/quasi-steady stages, during which the reaction slows down.

**Figure 9:**
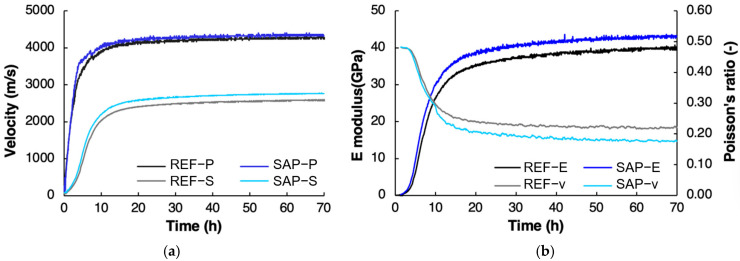
(**a**) UPV and (**b**) dynamic elastic properties of REF and SAP concrete. Measurements were conducted through the FreshCon device and dynamic elastic properties were calculated by the embedded SmartPick software V1.73 [31]. P = P-wave; S = S-wave; E = E-modulus; and v = Poisson’s ratio.

As hydration evolves, concrete undergoes hardening and the corresponding wave velocity increases with time, which explains the use of different curing timeframes in the localization algorithm. While parameters such as humidity gradients and temperature fluctuations influence wave propagation, their impact is secondary to the exponential gain in stiffness during concrete setting, which is primarily a topological effect [44]. To account for the hardening, this study adopts a time-dependent velocity model calibrated through experimental UPV measurements performed on the same composition, as shown in Figure 9. This ensures that localization results reflect the source activity considering the material’s stiffness development due to hydration. Figure 9 shows the measured UPV (P-wave and S-wave) as well as the deducted dynamic E-modulus and Poisson’s ratio for the studied SAP composition. REF is shown for completeness.

Localization of internal hydration sources allows the spatial distribution of AE events to be mapped, distinguishing between surface activity and events occurring deeper in the bulk. This capability also allows tracking of the temporal evolution of internal curing, showing how SAP activation progressed through the specimen. Furthermore, localization enhances the interpretation of the AE data by separating internal curing events from other early-age processes, such as shrinkage-related detachment from the formwork walls, thereby improving the reliability of the analysis and providing additional insight into the evolving mechanical properties of the material. Therefore, the evolving AE activity can be used to monitor the global process of internal curing, while acoustic localization can also shed light on where in the volume of the material this process is mostly active, information that cannot be obtained by any other measurement technique.

**Figure 10:**
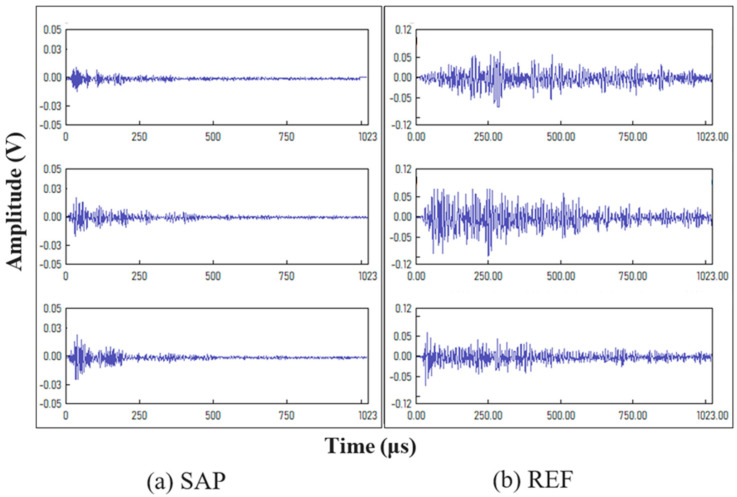
Indicative waveforms of (**a**) SAP and (**b**) REF concrete (from [45]).

Finally, indicative AE waveforms obtained from SAP detachment activity and from the REF concrete are shown in Figure 10 for completeness, as obtained from previous research [45]. The REF signals exhibit a clear increase in amplitude and duration, reflecting higher-intensity cracking signals, whereas the SAP detachment produces AE signals with lower values.

### 4.2. Localization Accuracy

It is very common in AE localization for events to overlap or occur close in time. Although this possibility cannot be fully avoided, it can be severely mitigated. To prevent multi-event mixing in the AET methodology, an event is accepted only if all eight sensors are triggered within a strict coincidence window, Δt. This window is a conservative travel-time bound derived from the maximum physically plausible straight path within the modeled volume (D_tot_ ≈ 286 mm) and curing stage-relevant velocities. Based on these, coincidence windows of 100 μs, 74 μs and 70 μs were adopted for the three curing stages, respectively. These values include a modest safety margin to account for timing uncertainties and material heterogeneity. Standard AE timing parameters (PDT, HDT, and HLT) further reduce reflections and repeated hits.

While stochastic interaction of multiple sources is always possible under high emission rates, the primary objective of this study is the localization of the hydration-activated zones and the tracking of global stiffness evolution rather than the validation of every individual micro-event. The strict eight-channel coincidence requirement significantly reduces the probability of multi-event merging within a 10^-4^ s window, ensuring a reliable dataset for tomographic reconstruction. Moreover, because elastic waves propagate faster in steel than in curing concrete, energy preferentially couples into the mold and travels as guided waves. This creates non-spherical wavefronts and premature arrivals at the sensors. These physics necessitate explicit modeling of the steel region (through VDM) and employment of the FMM, as conventional straight-ray assumptions cannot account for these nonlinear propagation paths. Moreover, because elastic waves propagate faster in steel than in curing concrete, the first arrivals can be dominated by energy that couples into the steel formwork and travels along this fast transmission path. If the formwork is neglected in the forward model, the predicted travel times become systematically slower than the observed arrivals. This provides explicit motivation for why the steel region is represented via VDM and the use of the FMM to compute curved first-arrival travel times in the heterogeneous concrete–steel system, rather than relying on straight-ray assumptions.

The resolution of AET depends highly, among other factors, on the sensor configuration. Although the eight-sensor array is relatively sparse for conventional tomographic reconstructions, the information content in the present passive AE tomography is analogous to the product of the recorded events and the sensors (N_events_ × N_sensors_). Since the hardening process generates hundreds of internal sources, the resulting high ray-path density stabilizes the inversion, allowing the adoption of a lower sensor number, as also stated in [37]. Synthetic sensitivity tests in [32] confirm that for datasets exceeding 600 events, an 8-sensor configuration achieves satisfactory velocity field recovery, with 12 sensors yielding only marginal improvements. It should be noted that the 5 mm computational grid spacing is a discretization parameter and does not represent the effective spatial resolution, which is governed by ray coverage. Consequently, the resulting tomograms are interpreted through coarse-scale spatial trends to track the global hydration evolution rather than localized microstructural features.

It is important to mention that no significant noise sources were identified in the experiments. In particular, electromagnetic interference and frictional noise due to thermal expansion of the steel formwork are excluded, as demonstrated in prior experiments conducted on an empty formwork subjected to oven heating [29]. Therefore, the AE activity recorded originates from the material and the processes within. Moreover, previous calibration experiments provide an indicative estimate of the achievable precision. In particular, air-hose calibration tests conducted in [46] showed that events occurring near the geometric center of the specimen could be localized with an error on the order of 5–10 mm. This increased accuracy is primarily attributed to the sensor configuration, as centrally located events are approximately equidistant from all sensors, thereby maximizing the sensor coverage.

### 4.3. Monitoring of Concrete–Steel Formwork Debonding Due to Shrinkage

**Figure 11:**
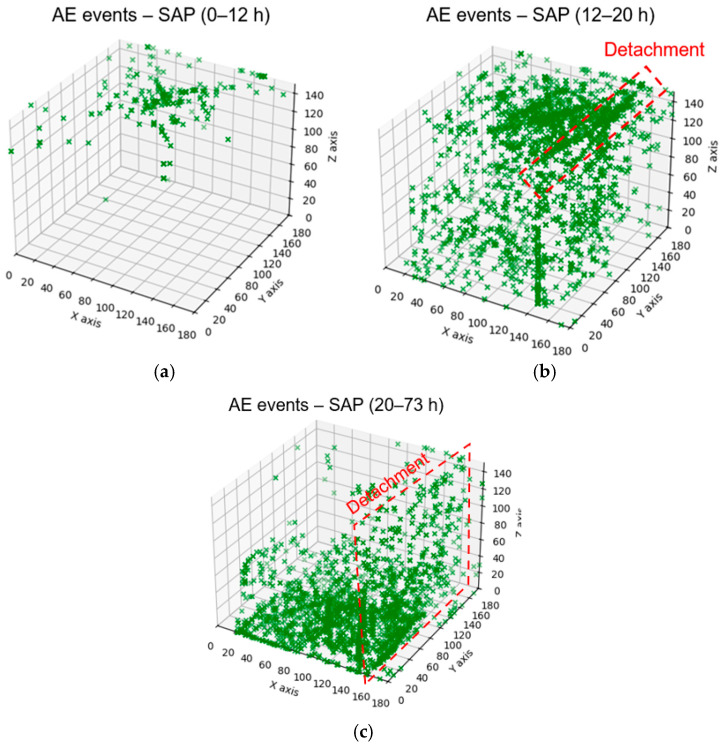
3D AET localization for SAP composition using the inhomogeneous model during (**a**) 0–12 h; (**b**) 12–20 h; and (**c**) 20–73 h.

**Figure 12:**
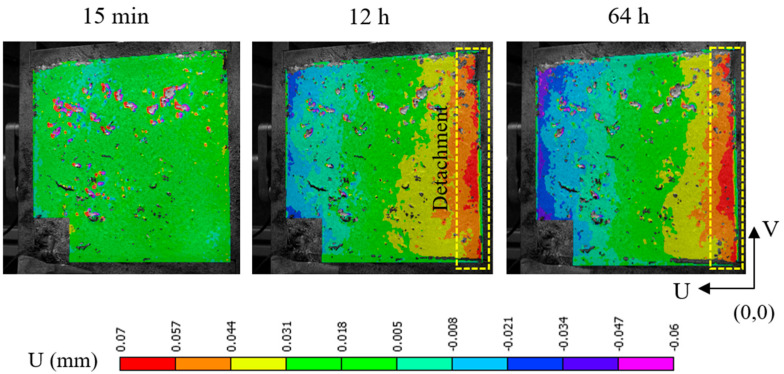
Visual representation of the surface displacement of hydrating SAP concrete, demonstrating the detachment in the concrete–formwork interface.

Acoustic localization supports characterization of the SAP activity and its progressive development deeper in the material volume, but also of other effects like debonding from the formwork wall due to shrinkage. Even though SAPs considerably mitigate the shrinkage, the latter cannot be fully avoided, particularly at the top surface when the concrete is exposed to the environment. During the 0–12 h curing stage, events are localized only at the material surface, indicating surface-level SAP activation, as in Figure 9a. Within the 12–20 h timeframe, several events are localized along the formwork side, as highlighted with red-dashed rectangles in Figure 11b, suggesting shrinkage-related debonding. This detachment continues during the 20–73 h timeframe, with events progressively moving towards the bottom of the formwork, as in Figure 11c. The detachment process was further confirmed visually by 3D Digital Image Correlation (DIC) performed on a secondary specimen of the same SAP composition, shown in Figure 12. DIC is a contactless technique that uses two cameras to track the displacements and strains on the surface of materials, through the application of a high-contrast speckle pattern, and has been effectively used to monitor fresh concrete and fresh concrete with SAPs in the past [47,48]. The surface displacement U during curing is evident on the specimen in the respective curing timeframes, supporting the detachment assumption. The aforementioned findings illustrate that acoustic localization, even in a material as heterogeneous and attenuative as fresh concrete, provides reliable information on mechanical processes like early-age shrinkage as well as microstructural processes like internal curing.

## 5. Conclusions and Outlook

This study localizes the hydrating sources in hardening SAP concrete using AE for the first time in the literature. The curing process was divided into representative curing phases, and different wave velocities were applied to account for the changing stiffness of concrete as it undergoes hydration. Traditional linear AE localization is compared to computed nonlinear localization obtained from AET. The influence of the steel formwork on localization accuracy is discussed. The following conclusions can be drawn from the results:

Internal curing unfolds progressively from the free surface downward, following the gradient created by drying. As moisture evaporates from the surface, it results in a drop in internal RH, guiding the curing front to develop from the drying top towards the protected bottom of the specimen. Although acoustic localization can take place even considering homogeneous conditions, it is significantly improved in the framework of AET that is capable of identifying different material properties. In this case, heterogeneities in the form of the two-phase material model (concrete–steel formwork) are accurately depicted, while a sufficient number of events is localized.

AE tomography provides useful insights into the stiffness development in the volume, while information on attenuation phenomena is expected to enhance accuracy during the initial fresh state. Another limitation of AE and AET source localization is that there is no particular way to validate the actual location of each one of the sources inside the material. In this respect, the comparison with visual information, as was supplied by DIC in the case of debonding, was important for the reliability of the acoustic localization. This study shows the potential of non-intrusive acoustic monitoring in characterizing complicated microstructural processes in fresh and hydrating concrete.

While the present study is limited to two specimens due to the computationally intensive nature of 3D tomographic reconstruction, the reliability of the findings is reinforced by the high degree of consistency between the two independent localization datasets. The AET results showed a direct physical correlation with both the localized cumulative AE activity and the visual displacement data captured via 3D DIC. Consequently, the results represent a robust internal validation of the AET method’s capability to monitor complex, nonlinear hardening processes, even within a focused sample set. Still, while the current findings offer significant proof-of-concept for monitoring the internal curing evolution, these conclusions should be further strengthened in future work by analyzing a larger number of specimens.

## Figures and Tables

**Table 1:** Concrete mixture composition (all units are in kg/m^3^).

Composition	C	S	G 7/14	G 4/8	Water	Additional SAP Water	SP	SAPs
SAP	388	496	496	922	136	16	2.33	0.78

**Table 2:** AE source localization setup.

Parameter	Value
Threshold (dB)	35
Preamplifier gain (dB)	40
Peak definition time (μs)	200
Hit definition time (μs)	800
Hit lockout time (μs)	1000
Overcall value (μs)	50
Sample rate (MSPS)	1
AEWin localization type	3D
Event definition value (mm)	370
Event lockout value (mm)	400
Overcall value (mm)	50
Min sensor number for event formation	6
Max iterations	256

**Table 3:** AE localization: 8-channel configuration (steel formwork neglected).

Channel	X (mm)	Y (mm)	Z (mm)
1	15	0	15
2	135	0	15
3	135	0	135
4	15	0	135
5	135	150	15
6	15	150	15
7	15	150	135
8	135	150	135

**Table 4:** Selected curing stages and corresponding elastic wave velocities for 3D source location.

Curing Stage	Timeframe (h)	UPV (m/s)
Early hydration	0–12	3000
Internal curing	12–20	4100
Hardening	20+	4300

## Data Availability

Data will be available upon request.
